# Mechanical and physical assessment of epoxy, mineral, solvent-based, and water-soluble coating materials

**DOI:** 10.1038/s41598-022-18022-0

**Published:** 2022-08-11

**Authors:** Ginneth Patricia Millán Ramírez, Hubert Byliński, Maciej Niedostatkiewicz

**Affiliations:** Department of Engineering Structures, Faculty of Civil and Environmental Engineering, Gdansk University of Technology, 11/12 Narutowicza street, 80-226 Gdańsk, Poland

**Keywords:** Engineering, Materials science

## Abstract

This paper assesses the behavior of mineral, epoxy (EP), solvent, and water-soluble coatings when exposed to salt and regular water for 28 days. Also, it evaluates the *pull-off* adhesion strength of the same coating materials applied to concrete slabs saturated with oil and water and dried with two different processes: air-dried for 28 days and air-dried for 14 days plus 14 days in the oven at 70 °C. Properties such as carbonation, water absorption rate, *pull-off* adhesion strength were evaluated for all coatings, and tensile strength, Young’s modulus, and elongation percentage were calculated for mineral coatings. According to the results, the EP coating showed the best performance with the highest *pull-off* adhesion strength (2.55 MPa) and lowest absorption rate, about 0.02 ± 0.002 g/m^2^ day in saltwater and 0.03 ± 0.002 g/m^2^ day in regular water. In addition, EP coatings also presented the lowest carbonation rate and the highest suppress ratio. The excellent performance of epoxy coatings is mainly due to their low porosity and the ability to decrease chloride diffusion, making them better than other types of coatings investigated in this study.

## Introduction

Different atmospheric conditions can highly affect concrete durability. Among the most common durability problems, carbon dioxide increases the carbonation depth causing fatal concrete deterioration and corrosion of steel reinforcement^1–3^. Different researchers have studied carbonation and have proposed prediction models to analyze and understand carbonation phenomena in different environments^4^. For example, Park^5^ presented a carbonation model considering the diffusion of carbon dioxide on concrete samples protected with coating materials. As a result, polyvinyl chloride coating has a smaller diffusion coefficient than epoxy and acrylic coating, meaning that polyvinyl chloride coating decreases the carbonation depth over time. In another case, Cunha Reis et al.^6^ assessed the efficiency of traditional cementitious-based coatings for concrete protection against carbonation by using an accelerated method, achieving a 56% reduction in carbonation depth compared to concrete samples uncoated. Results suggest that coating materials protect concrete and reduce carbonation depth regardless of their composition.

**Figure 1:**
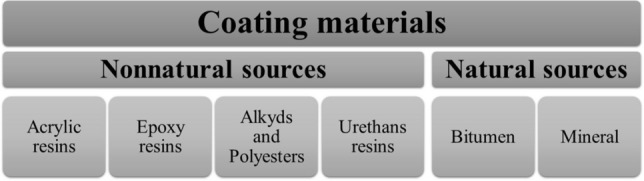
Most common coatings materials used for protecting concrete structures^9–12^.

Since prehistory, coating materials have been used to protect and decorate elements and surfaces^7^. During the last decades, different compounds have been used to manufacture coating materials, starting from basic synthetic resins to developing high-performance resins^8^. The construction industry uses several of these materials to protect concrete structures against corrosion. Figure 1 describes some of the most common coatings materials used in concrete structures.

Coating materials used to protect construction elements embedded into the ground are more popular than geotextiles because of the ease of application and the lower cost of material and labor. However, nowadays, one of the major limitations of coating materials is their application on surfaces saturated with water or oil during the construction process or when the structures are previously exposed to different contaminants.

Different studies used coating materials to improve the concrete surface and minimize the effect of corrosion. For example, Aguirre-Guerrero et al.^13^ assessed the effectiveness of coatings based on alkali-activated and epoxy materials to protect concrete samples when exposed to chlorides. The outcomes showed that epoxy and inorganic coatings presented the lowest capillary absorption coefficient. Epoxy material is characterized by its physical barrier, which makes the material limit the diffusion of chlorides by obtaining the lower current values among all coatings, showing a significant increase in the material resistivity. Suleiman et al.^14^ assessed the performance of bitumen-modified polyurethane coating when concrete samples are exposed to sulfate attacks. This coating shows similar properties than epoxy coating regarding the low sulfate penetration due to the thick membrane that forms on the surface of the concrete. However, it is important to highlight that this material requires proper curing of the concrete before the application since separations of the coating with the concrete sample may occur. Diamanti et al.^15^ evaluate cement-based coatings’ water absorption, permeability, chloride penetration, and coating adhesion properties. It is evidenced that cement-based coatings slow down the chloride penetration and reduce the water content compared with samples without coating protection. Additionally, pull-off strength is affected by the W/C ratio; with lower W/C ratios, there is a decrease in the concrete porosity, which benefits the pull-off adhesion strength results. Finally, Krzywinski et al.^16^ intended to improve the pull-off adhesion strength of epoxy coatings by texturizing the concrete surface with different imprinting, grabbing, and brushing methods. The results indicate that the pull-off strength could increase by about 9% when plastic cross shapes are used compared to the manufacturer’s recommendations.

Bitumen, epoxy, and mineral coatings have been used more frequently in the construction sector to protect floors, pipelines, and different concrete elements^17^. To ensure that the material is suitable for protecting concrete elements is necessary to assess some of its mechanical and physical properties. Among the essential properties of coating materials are; adhesion to the substrate, absorption rate, tensile strength, and carbonation resistance^18,19^. The test methods used in the present research include: (1) Pull-off adhesion strength according to PN-EN 14,891:2017^20^ (2) Absorption test by immersing the samples in regular and saltwater (2% NaCl solution in water) for 28 days (3) Tensile strength according to ISO-527-2012^21,22^. (iv) Carbonation depth according to^23^. These measures were suitable for the four coating materials used during the experimental part.

This research aims to evaluate the influence on the mechanical and physical properties of the mentioned coating materials when the concrete surface is exposed to external factors such as oil and water. In addition, it assesses the influence of chlorides in coating materials samples and the carbonation resistance of concrete specimens protected by MI, EP, BR, and BW coatings. Experimental tests were carried out according to our own research procedures.

## Experimental procedure and materials

The methodology process involved different stages. First, the coating materials were selected based on their main compounds, i.e., epoxy, asphalt, mineral, and water. Afterward, coating samples were used to test their mechanical and physical properties. Tensile strength was measured just for the mineral coating by preparing samples of (50 × 250) mm. For absorption tests, 20 samples of each coating were exposed to salt and regular water for 28 days.

**Figure 2:**
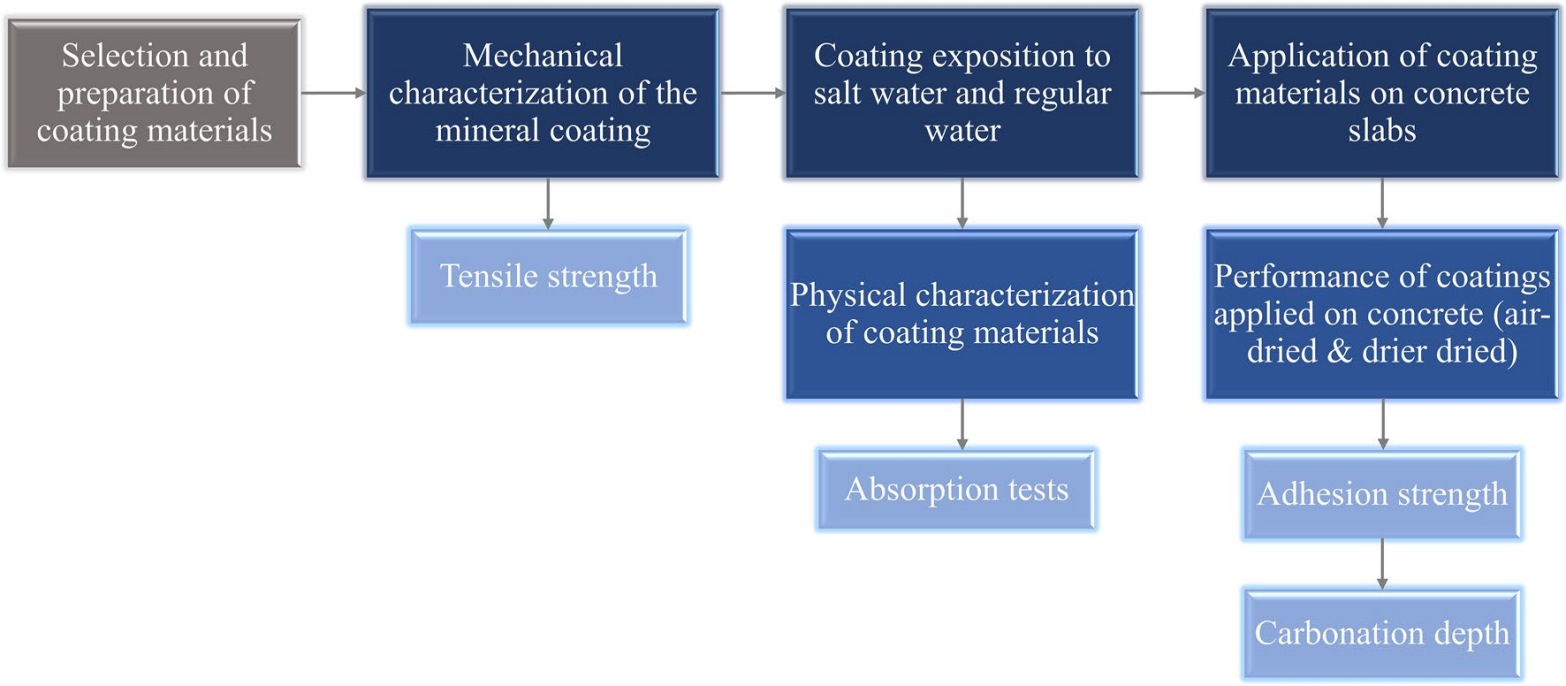
Methodology process used.

To assess the pull-off adhesion strength, mineral (MI), epoxy (EP), solvent-base (BR), and water-soluble (BW) coating materials were placed in concrete slabs and divided into two groups: In the first group, the coating materials were air-dried for 28 days; in the second group, the coating materials were air-dried for 14 days, plus, 14 days in a drier at 70 °C. Both groups used water and oil to impregnate the concrete surface to assess the coating material’s pull-off adhesion on contaminated surfaces. Figure 2 describes the stages used in this study.

### Materials

In this research, four types of coating materials commonly used in the construction sector for protecting underground concrete structures exposed to moisture were used: Epoxy coating (EP), mineral coating (MI), water-soluble coating (BW), and solvent-based coating (BR). Tables 1, 2, 3 and 4 describes the technical data of the coating materials used.

**Table 1:** Technical data of the commercial epoxy coating.

Composition	Density	Tearing resistance	Compressive strength (after 28 days)	Tensile strength (after 7 days)
Epoxy resin and polyamide hardener	1.10 ± 0.10 g/${\mathrm{cm}}^{3}$	2 MPa	≥ 80 MPa	≥ 25 MPa

**Table 2:** Technical data of the commercial Mineral coating.

Composition	Initial adhesion	Adhesion after thermal aging	Water pressure resistance	Capillary absorption and water permeability
Polymer-modified cement mixture	≥ 0.5 MPa	≥ 0.5 MPa	1.0 MPa	< 0,1 kg/${\mathrm{m}}^{2}$*${\mathrm{h}}^{0.5}$

**Table 3:** Technical data of the commercial solvent-based coating.

Composition	Viscosity, flow time at temperature (23 ± 0.5 °C)	Water content	Drying time
Industrial asphalt, organic solvent, additives, SBS	From 30 to 150 s	≤ 0.5% (m/m)	≤ 12 h

**Table 4:** Technical data of the commercial water-soluble coating.

Composition	Density	Water content	Content of non-emulsified asphalt
Water dispersion of asphalts, rubbers, and improvers	1.05 ± 0.05 g/${\mathrm{cm}}^{3}$	≤ 50% (m/m)	≤ 1.2% (m/m)

Additionally, concrete slabs of (350 × 350) mm, (200 × 200) mm, and (140 × 200) mm type B30 were used to perform adhesion tests. To determine the carbonation depth, a solution with 1% of phenolphthalein in 70% ethyl alcohol was used^23^.

#### Application of coating material

The pull-off adhesion strength was assessed under different conditions: dry, wet, and oily substrate. Before the coating application, the wet and oily substrate was simulated by painting the concrete slabs with two layers of water and industrial oil.

**Figure 3:**
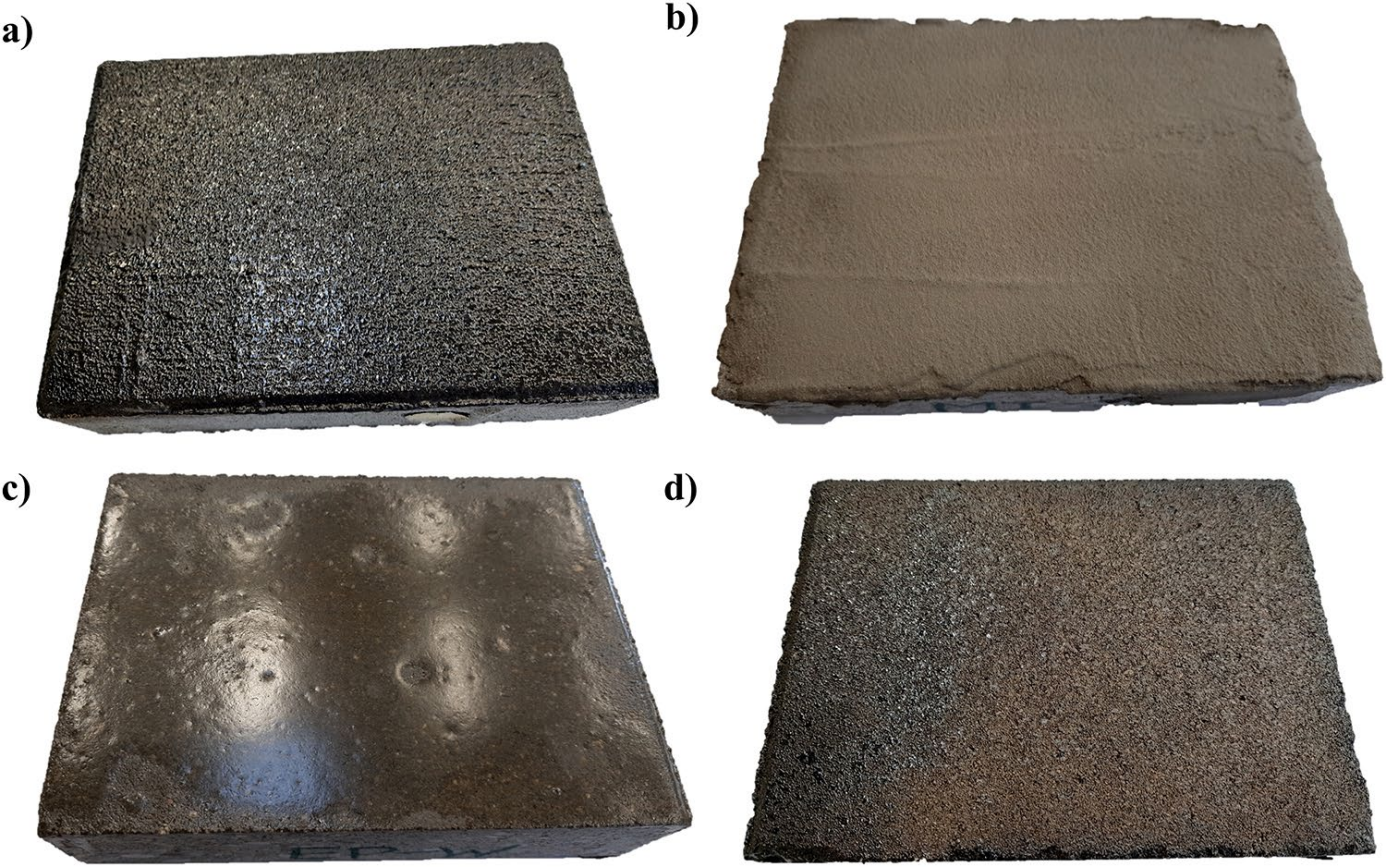
Coating materials applied in concrete slabs with wet substrate (**a**) BW (**b**) MI (**c**) EP (**d**) BR.

BW, MI, EP, and BR coating materials were applied according to the manufacturer’s specifications. A total of 36 concrete specimens were used during the research. Figure 3 shows the coating materials applied to concrete slabs.

The application process of the coating materials is described below.

- *Concrete surface preparation* A regular brush was used to clean the surface from dust particles.
- *Impregnation of the concrete surface with oil and water* Potable water and synthetic oil for car engines (type 5 W-40) was used to saturate the concrete surface’s superficial pores immediately before applying the coating materials.
- *Preparation of coating materials* There was no need for any preparation in the water-soluble coating and solvent-base case. These materials come ready to be applied. However, the epoxy resin had to be mixed with polyamide hardener for 8 min before application, and mineral coating needed to be prepared with 20% of water relative to the weight of the used material.
- *Application of coatings to the surface of the concrete slabs* The application varied according to the type of coating material.
  - *Epoxy coatings* After mixing the coating compounds, one layer of the mix was applied with a regular brush on the surface of each concrete sample.
  - *Water-soluble and solvent-based coatings* According to the manufacturer’s recommendations, three layers of the material were applied using a regular brush. The second and third layers were applied after the previous layer dried to touch, about 30 min for solvent-based coating and about 1 h for water-soluble coating.
  - M*ineral coating* After mixing the material with water, two layers were applied with a spatula, distributing the material uniformly on the concrete slab’s surface. The second layer was applied after the first layer had dried to touch after about 1.5 h.

After the application of the coating materials, the concrete samples were divided into two drying groups: in the first group, the concrete samples remained at room temperature of 20 °C ± 1 °C for 28 days, and in the second group, concrete samples remained at room temperature of 20 °C ± 1 °C for 14 days, followed by 14 days of drying in an electric drier.

### Experimental tests

#### Pull-off adhesion strength of coating materials

**Figure 4:**
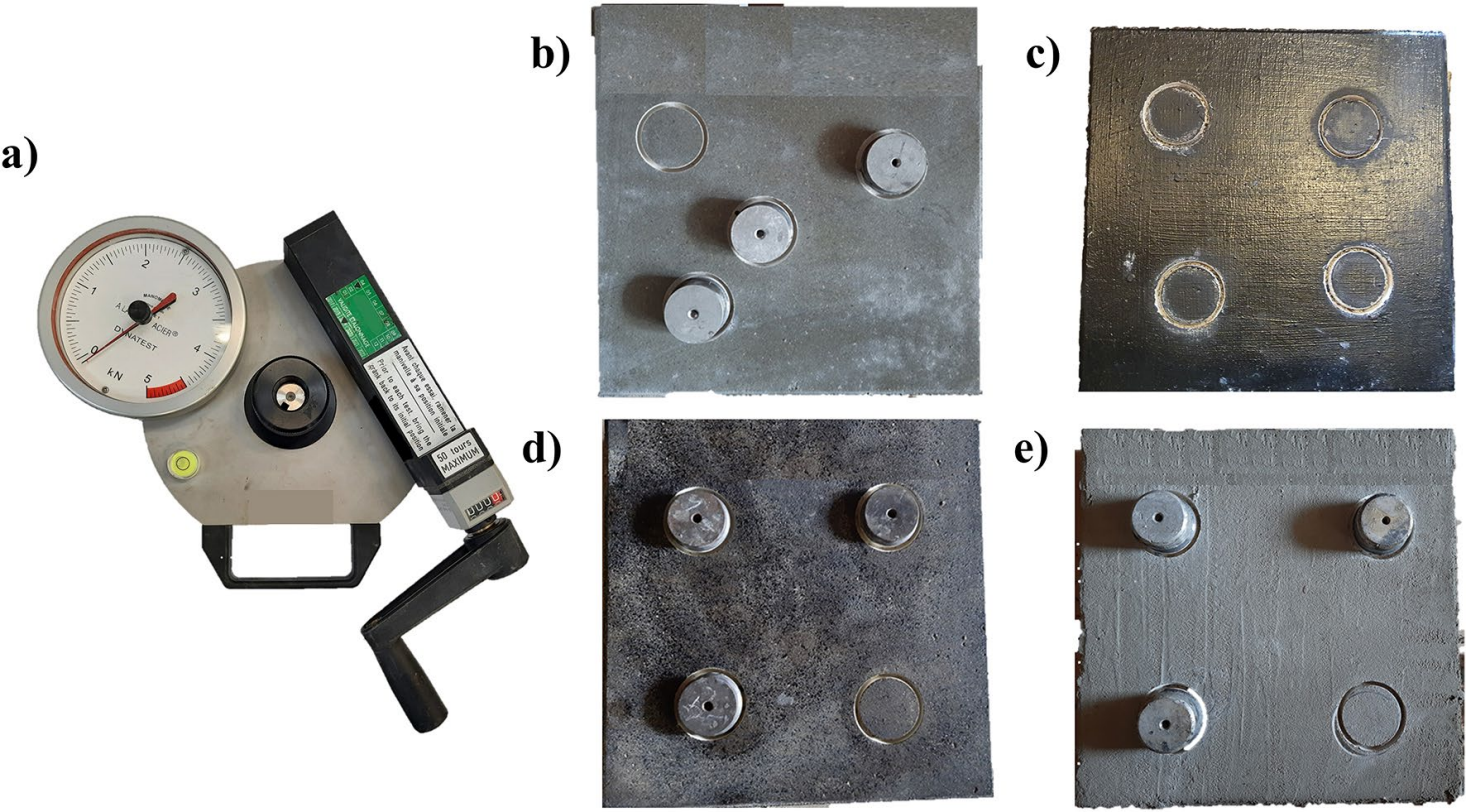
(**a**) Equipment used. Concrete slabs preparation for pull-off adhesion test: (**b**) EP (**c**) BW (**d**) BR (**e**) MI.

A traction dynamometer with a maximum tensile strength of 5 kN was used to perform the pull-off adhesion test. Before placing the metallic discs (dollies) on the concrete surface, it was necessary to drill a circular notch to define the measured surface accurately. Afterward, 50 mm-diameter/20 mm-height dollies were glued to the surface using a standard epoxy-based adhesive. Figure 4 shows the equipment used to perform the tests and some examples of concrete slabs with the dollies. A total of 70 pull-off tests were taken. The Pull-off strength test was performed according to PN-EN 14,891:2017 standard^20^ and ASTM D 4541 standard^24^.

#### Carbonation depth

After applying the coating material, concrete samples were stored in an indoor controlled environment at 20 ± 2 °C and 75% ± 5% relative humidity for 90 days. Next, phenolphthalein solution was sprayed onto the fresh broken concrete surface according to the CPC-18 RILEM^23^. This solution is characterized by being a colorless liquid commonly used as an acid-base indicator that changes to purple when the pH is higher than 9. As a result, the non-carbonated concrete surface, where the specimen is still alkaline, turns into a purple color, and the carbonated concrete surface remains colorless due to the reduction in the alkalinity of concrete.

**Figure 5:**
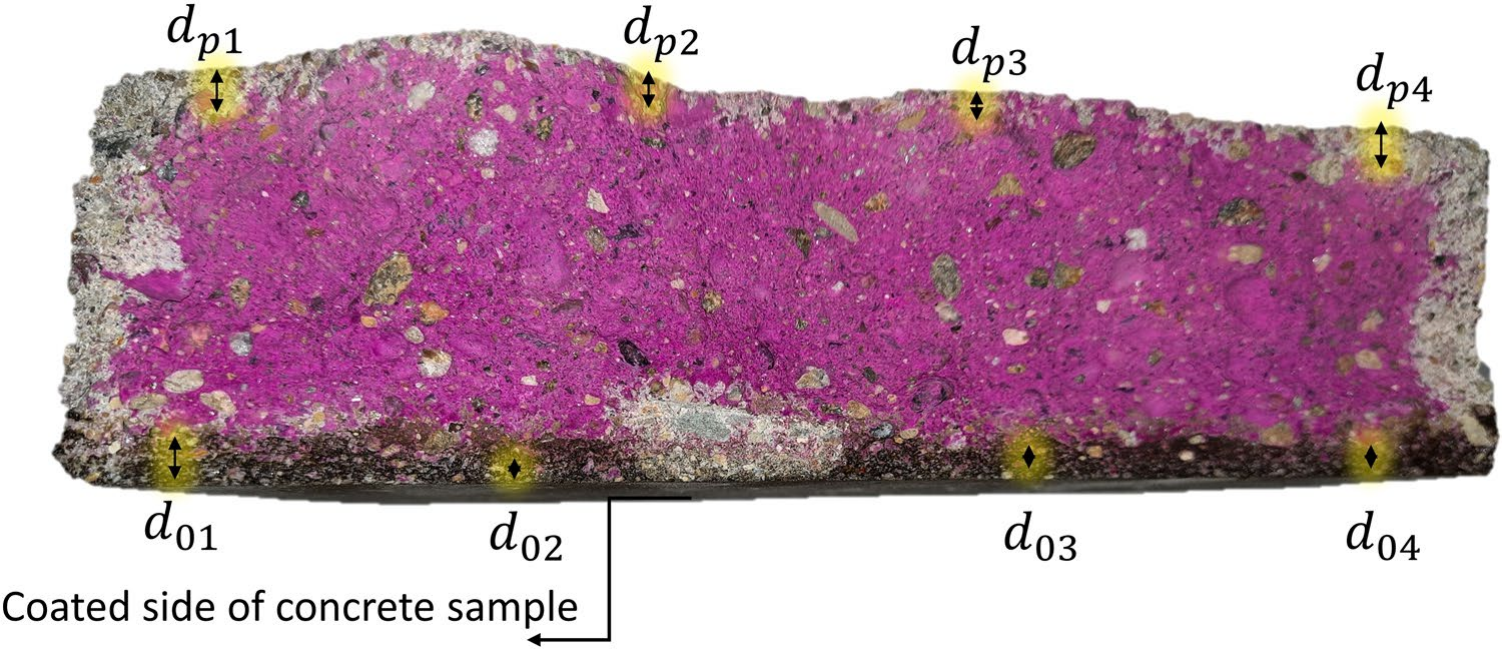
Carbonation measurement on both sides of concrete samples.

According to Page et al.^25^, the performance of coating materials against corrosion can be measured by the carbonation suppression ratio ($${R}_{cs})$$ which compares the coated and the uncoated sides of concrete samples. Figure 5 shows the average depth $${d}_{0}$$ and $${d}_{p}$$.

#### Absorption test

**Figure 6:**
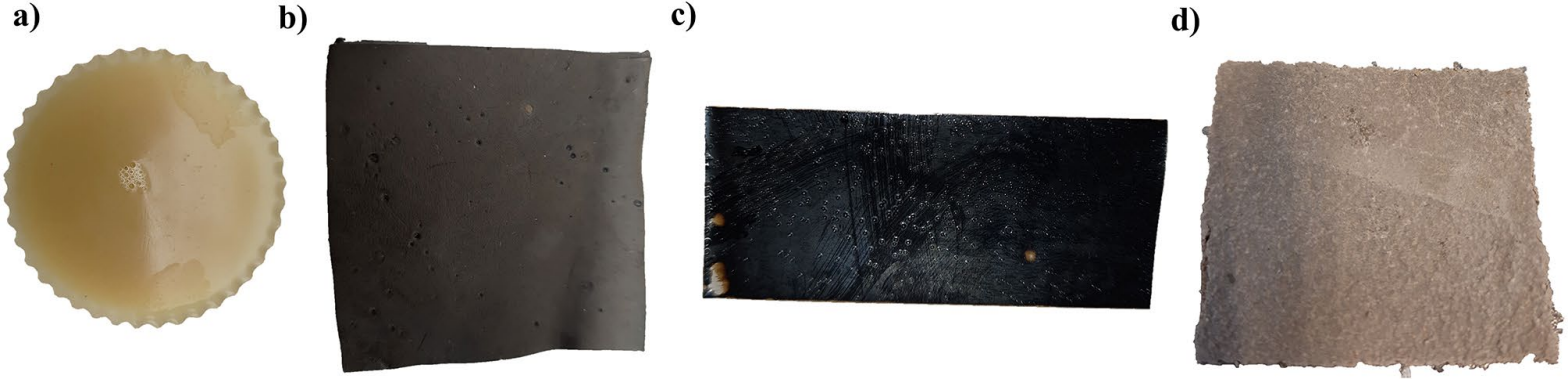
Coating materials samples. (**a**) EP (**b**) BW (**c**) BR (**d**) MI.

Absorption was measured by weighting the different coating samples after 28 days of exposition to salt and regular water. First, each coating was applied on silicone foils and was air-dried for seven days in a controlled environment at 20 ± 2 °C and 75% ± 5% relative humidity. Then, mineral and water-soluble coatings were cut to size (30 mm × 30 mm), and some individual coating samples were made for epoxy and solvent-based types. Figure 6 shows the coating samples used to test absorption. Equations proposed by Almusallam et al.^26^ were used to calculate the water absorption rate.

#### Tensile test of the mineral coating

Tensile strength in mineral coatings was assessed by using samples of size (250 mm × 50 mm) and conditioned at 20 ± 2 °C and 75% ± 5% relative humidity. Tensile tests were performed at 14 and 28 days of air-drying using a testing machine with a maximum test load of 5 kN. The free distance between the holders was about 205 ± 1 mm, and the transverse speed was set to 10 mm/min. A total of eight specimens were tested. Elastic modulus, tensile strength, and strain at fracture in tension were calculated at the maximum load.

The samples were prepared by bonding end-tabs of epoxy tape, resulting in an available cross-area of approximately 60 mm^2^.

### Research limitations

This paper was limited to English articles found in the following journals: Elsevier “Construction and building materials,” Springer “Journal of Materials Science,” MDPI “buildings,” MDPI “Coatings,” which excludes literature published in other languages and is also limited to academic publications. Moreover, it does not consider the results from industrial practice.

## Results and discussion

### Pull-off adhesion strength of coating materials

**Figure 7:**
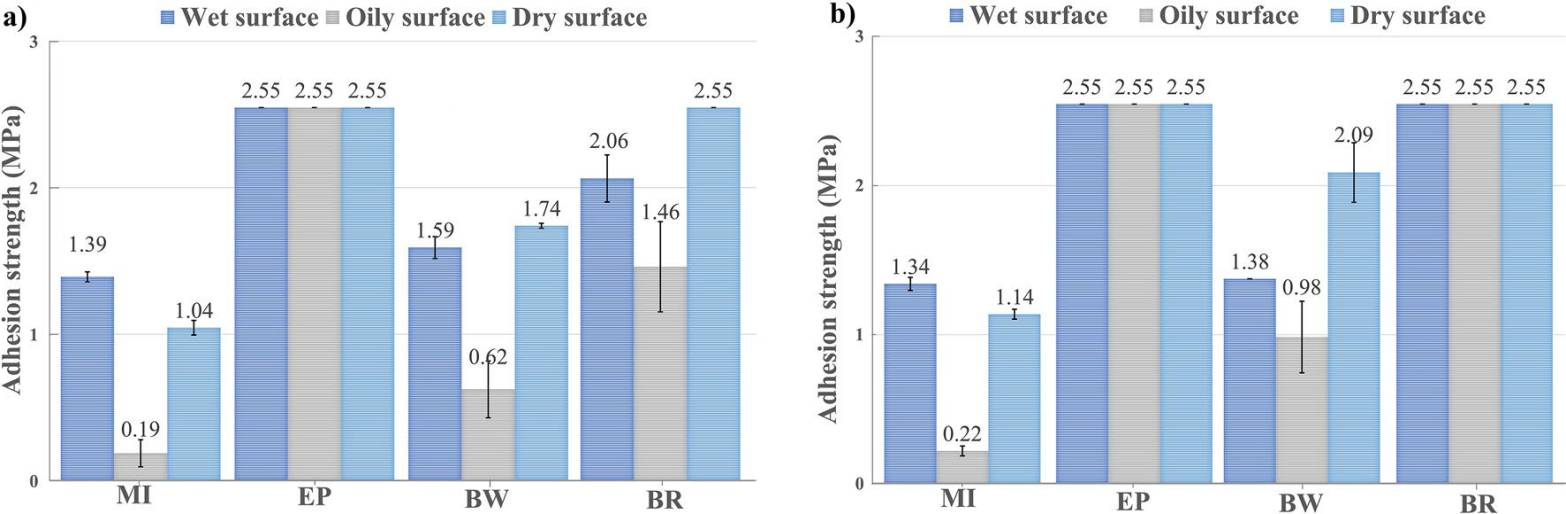
Average adhesion test in concrete slabs (**a**) Samples air-dried for 28 days (**b**) Samples air-dried for 14 days + oven-dried for 14 days.

Figure 7 shows adhesion strength values for MI, EP, BW, and BR coatings applied on concrete slabs and dried in two conditions and on three surfaces. [Point 2.1.1.].

Only the EP coating products presented physical changes after applying the different coating materials on the concrete slabs. In the oily substrate, it was evidenced that epoxy coating could not interact with the oil molecules, making the coating repelling and leaving certain areas without coating material. In the case of wet and dry substrates, no physical changes were evidenced. Figure 9 shows the EP behavior after the application on oily, wet, and dry substrates.

**Figure 8:**
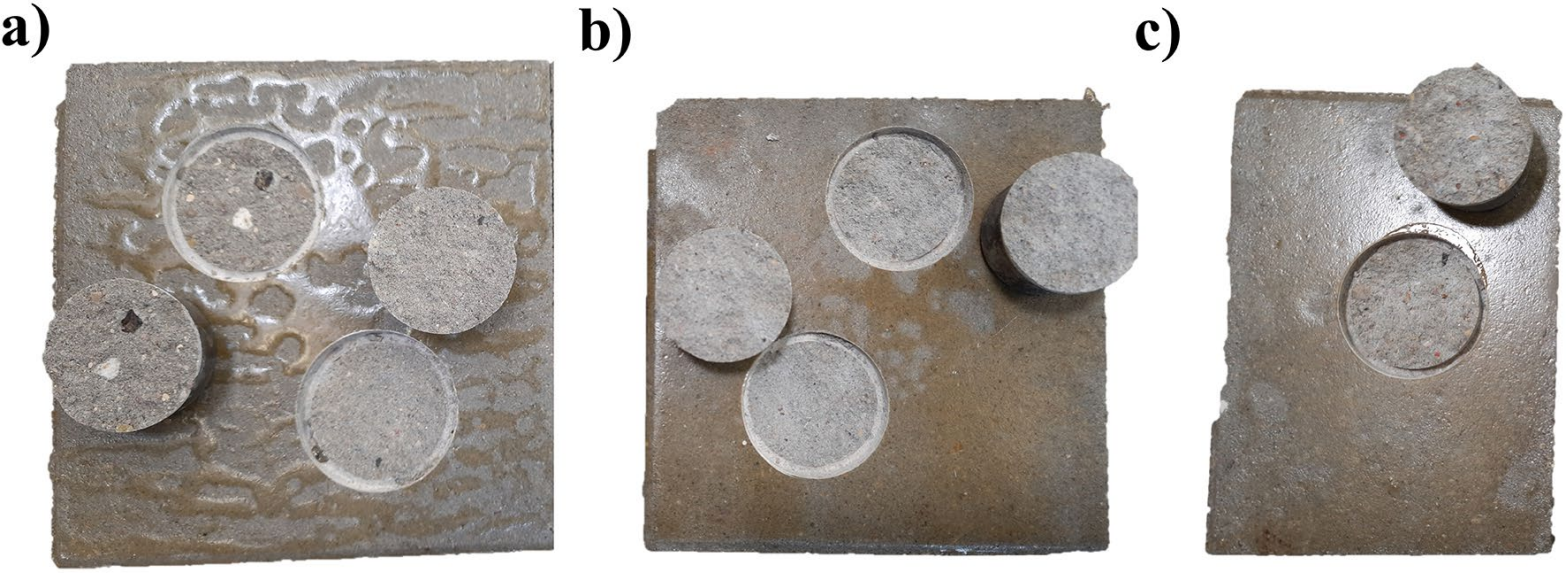
Adhesion tests in concrete samples with EP coating after 28 days of air-drying: (**a**) Oily substrate (**b**) Wet substrate (**c**) Dry substrate.

The results indicate that epoxy materials presented the highest adhesion strength values in all scenarios by exceeding the maximum possible measure of the dynamometer (5 kN). These values follow the values given by the manufacturer. It is noteworthy that all EP samples presented substrate failure under the load, evidencing a concrete substrate strength weakness. Figure 8 shows concrete slabs painted with EP after being subjected to the pull-off test.

**Figure 9:**
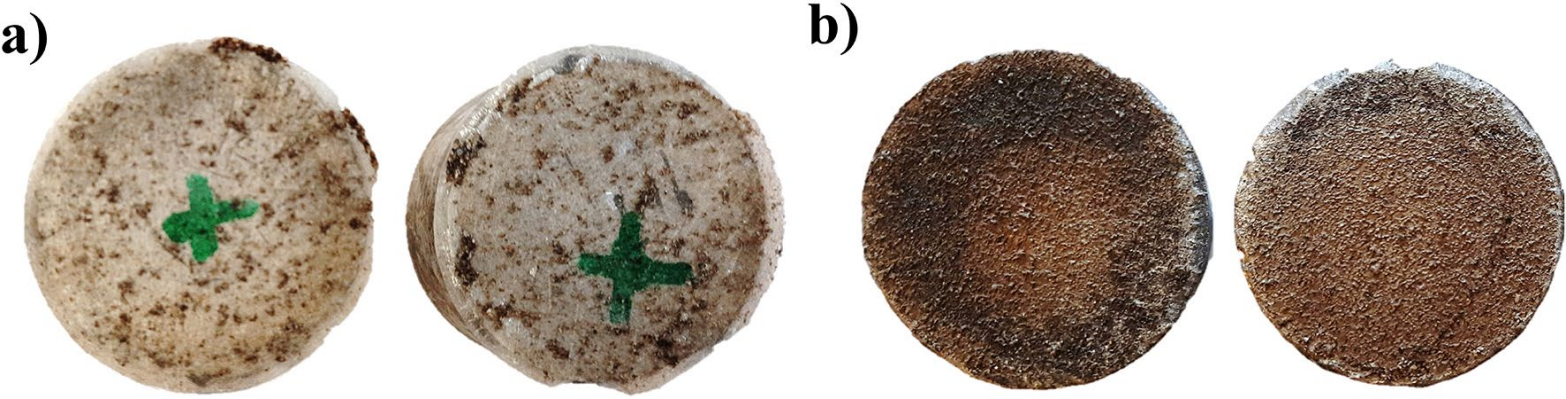
Dolly after pull-off adhesion test in the dry substrate: (**a**) BR samples exposed to air and oven drying (**b**) BR samples exposed to air drying.

For concrete slabs with BR Coating, the maximum pull-off adhesion strength values were observed in the samples dried for 14 days in the air plus 14 days in the oven by achieving the maximum pull force value (5 kN). However, according to the types of failures presented in the ASTM D 4541 standard and ISO 4264^24,27^, a glue failure was evidenced in all samples; this can be attributed to the drying process in the oven. The coating reduced its mechanical properties and did not chemically interact with the epoxy resin, leaving a weak joint. For samples exposed to air drying for 28 days, a reduction in the strength adhesion was evidenced; these specimens showed a decrease of 42% in samples saturated with oil and 19% in samples saturated with water. Even though there is a reduction in the strength values, the specimens presented a cohesive failure within the coating layer, which is considered the ideal type of failure in the pull-off test. Figure 9 shows examples of the types of failures mentioned for BR coatings.

**Figure 10:**
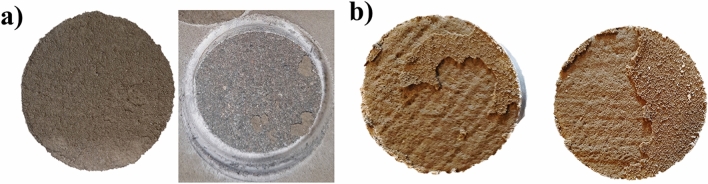
Mineral coating in the oily substrate: (**a**) MI samples exposed to air and oven drying (**b**) MI samples exposed to air drying.

**Figure 11:**
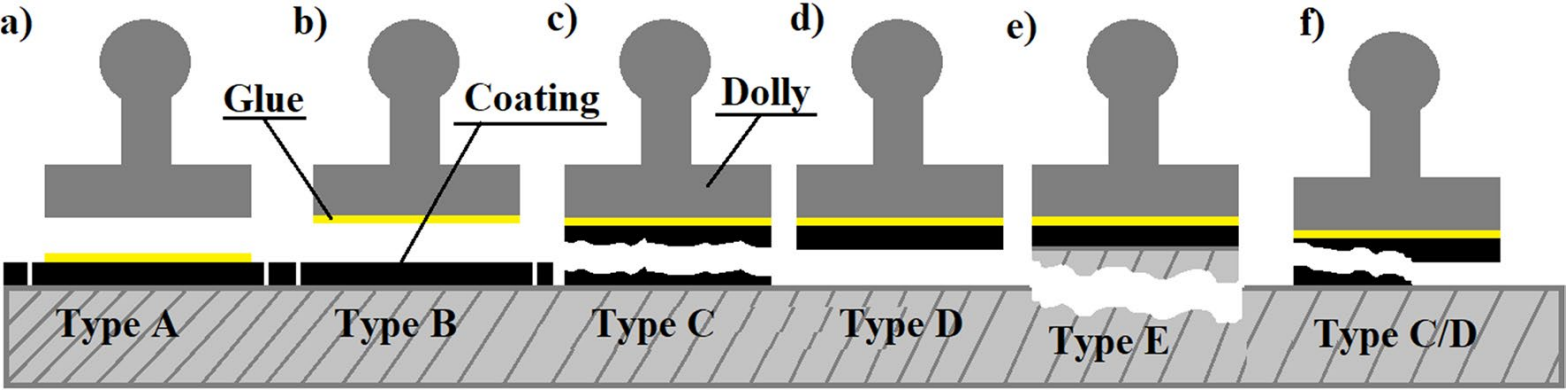
Types of fracture according to its nature: (**a**) Glue failure (**b**) Adhesive failure (between layers) (**c**) Cohesive failure (within a layer) (**d**) coating failure (**e**) Substrate failure (**f**) 50% cohesive failure and 50% coating failure.

The mineral coating was the only material where the presence of oil influenced the failure type. Samples exposed to air-drying presented 50% adhesive and 50% (Fig. 11b) cohesive failure, and samples exposed to oven drying showed 100% adhesive failure (Fig. 11a). For wet and dry surfaces, cohesive failure was evidenced in both types of drying, showing values aligned to those of the manufacturer. Figure 10 illustrates concrete specimens saturated with oil surface and protected with a mineral coating.

The BW coatings presented 100% cohesive failures in all samples. The results show a slight increase in *pull-off* adhesion strength for the specimens subjected to oven drying.

ISO 4264^27^ was used to assess the fracture type. Figure 11 describes the nature of the possible fractures.

According to Fig. 11, Table 5 summarizes the types of fracture obtained in all pull-off adhesion strength tests.

**Table 5:** Adhesion test summary of types of failure.

Type of coating	Air-dried			Air-dried + Oven-dried		
	Wet surface	Oily surface	Dry surface	Wet surface	Oily surface	Dry surface
MI	C	C/D	C	C	D	C
EP	E	E	E	E	E	E
BW	C	C	C	C	C	C
BR	C	C	C/E	B	B	B

Among the results, the decrease in the pull-off adhesion strength was mainly evidenced in concrete samples saturated with oil. The presence of oil reduced the strength values for MI coating by 84% and 81% compared to wet and dry surfaces; this made the results invalid since they did not comply with the ASTM D 4541 standard^24^ range established between 1.37 and 3.45 MPa. BW coating also showed a reduction in the pull-off adhesion strength of 44% and 59% for the wet and dry surfaces. Again, similar to MI, these results were invalid due to non-compliance with the standard.

### Carbonation depth

Based on the previous results, the air-dry method and the samples saturated with regular water were selected as optimal samples because their physical and mechanical properties were not widely affected. The average carbonation depth and the suppression ratio were calculated to assess the reduction in the carbonation caused by the application of MI, EP, BW, and BR materials. Table 6 shows the average carbonation depth and the suppression ratio for MI, EP, BW, and BR coatings after 90 days of standard curing.

**Table 6:** Average carbonation depth and suppression ratio.

Coating material type	Surface type	Average carbonation depth and suppress ratio
MI	Coated	8.45 ± 2.44 mm
	Uncoated	8.73 ± 2.29 mm
	Suppression ratio (%)	3.15%
EP	Coated	1.98 ± 0.67 mm
	Uncoated	4.63 ± 1.48 mm
	Suppression ratio (%)	57.30%
BW	Coated	1.88 ± 0.61 mm
	Uncoated	3.48 ± 1.24 mm
	Suppression ratio (%)	46.04%
BR	Coated	2.40 ± 0.75 mm
	Uncoated	3.23 ± 1.40 mm
	Suppression ratio (%)	25.58%

A notorious carbonation depth reduction in samples protected with EP coating was evidenced, going from 4.63 mm to 1.98 mm (57% reduction) compared to MI coating, which only presented a drop of about 3%. EP coatings have shown adequate concrete protection against carbonation compared with other materials such as polyurethane, acrylic, and chlorinated rubber when exposed to accelerated aging methods^2,28^.

On the other hand, water-soluble coating presented a higher suppression ratio compared to solvent-based coating, approximately 20% higher; this is mainly due to the evaporation of solvent components during the oven’s drying process, causing a decrease in the protection of concrete against carbonation.

### Absorption test

**Figure 12:**
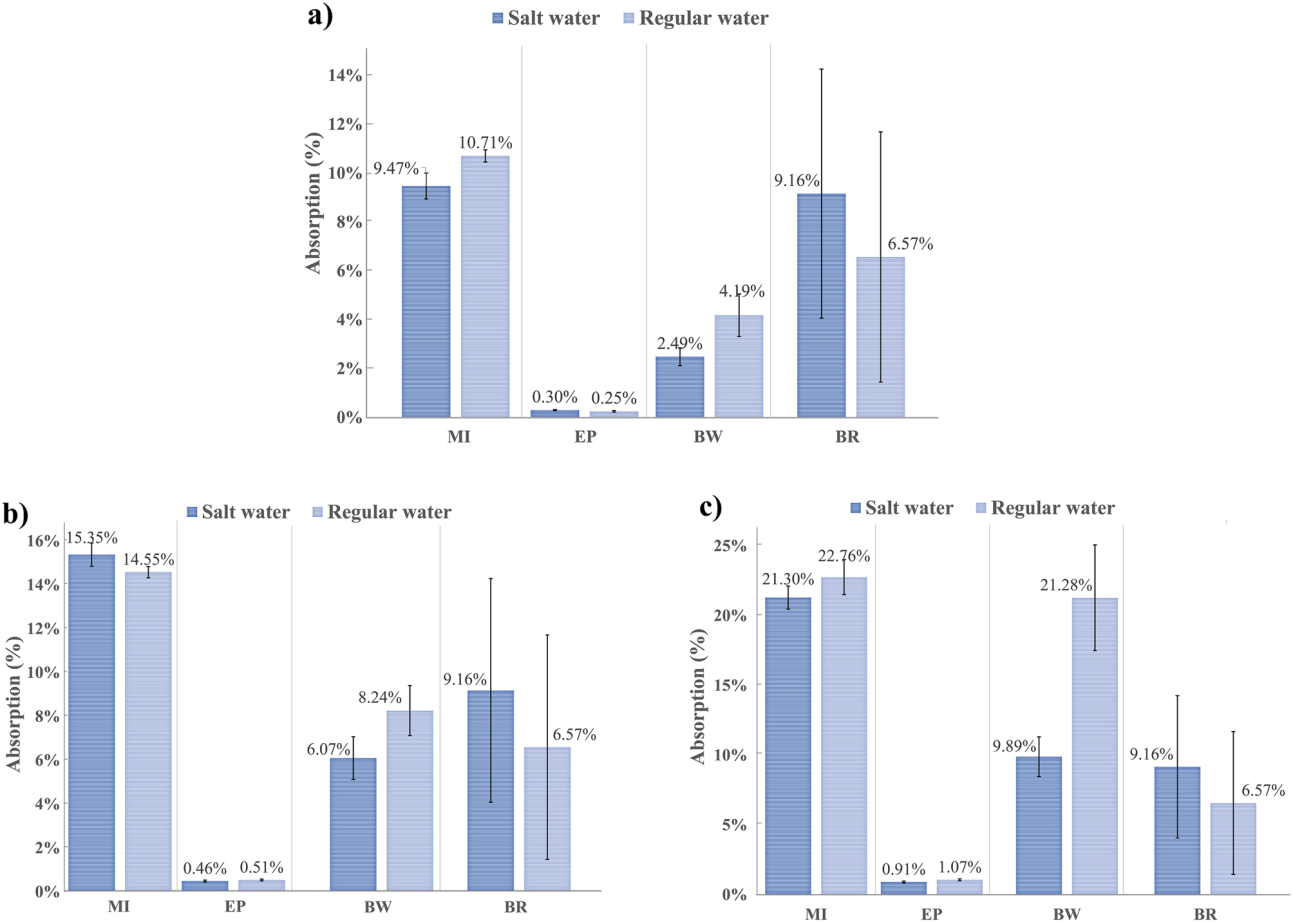
Average coating water absorption: (**a**) After 24 h (**b**) After 7 days (**c**) After 28 days.

Figure 12 shows the results of the water absorption test of the four coating materials at different stages, 24 h, 7 days, and 28 days. Table 7 describes the final weight gained by the different coating materials samples.

**Table 7:** Average weight gain by MI, EP, BW, and BR coatings after 28 days if exposition to salt and regular water.

Type of coating	Saltwater	Regular water
MI	21.30% ± 0.82%	22.76% ± 1.26%
EP	0.91% ± 0.06%	1.07% ± 0.07%
BW	9.89% ± 1.43%	21.28% ± 3.79%
BR	9.16% ± 5.10%	6.57% ± 5.12%

**Figure 13:**
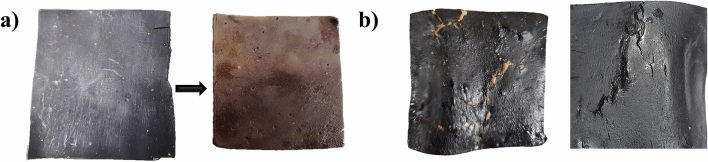
Physical changes in BW coating after 28 days of exposition (**a**) Produced by exposition to regular water (**b**) Produced by exposition to saltwater.

According to the results, MI, EP, and BR coatings presented similar absorption coefficients between each of them during the three stages when exposed to regular and saltwater. However, BW coating was widely affected by regular water, showing a total increment in the absorption coefficient after 28 days of exposition of around 21% in regular water, 10% more than in saltwater. According to Anderson et al.^29^, the low water absorption is caused by hydrocarbon constituents, showing a slight tendency to absorb water over the years. Additionally, physical changes were noticeable after seven days of contact. In the case of regular water, changes in the sample color were evident due to the chemical changes produced by the presence of oxygen that interacts with the basic components of the material, changing from black to brown color. For saltwater, some samples showed cracks in their surface, accelerating the corrosion of specimens. Figure 13 shows the changes in the color of the coating samples.

It is noteworthy that EP coating presented the lowest water absorption values during the exposition time attributable to its low shrinkage performance by controlling expansive chemicals and minimizing cracks due to internal stresses. As a result, the samples showed a smaller gain in weight and a maximum absorption value of 1% after the 28th day, which was aligned with the manufacturer’s specifications. There is no evidence of physical change in the presence of regular or saltwater for the EP samples. In contrast, since the initial stage, the MI coating showed the highest absorption results among all samples during the exposition time, increasing from 9.47% after 24 h of exposition to 21.30% on the 28th day for saltwater. And from 10.71 to 22.76% for regular water.

Furthermore, after seven days of exposition, the MI coating presented small filaments perpendicular to the surface of the specimen; this appears to be an accumulation of salts. In the case of regular water, any physical change was evidenced.

It is important to mention that BR samples did not change in weight after 24 h of exposure. Therefore, water absorption occurred only during the first 24 h; however, the specimens exposed to regular water showed the appearance of apparent bubbles on the surface. According to^29^, this material has a significant amount of hydrocarbons in its chemical composition, which decreases the tendency to absorb water. However, asphalt materials may contain inorganic salt in small proportions that could promote water absorption when moving through the membrane.

Data presented in Fig. 12 was used to calculate the water absorption rate, and Table 8 shows the results.

**Table 8:** The average absorption rate for the different coating materials in salt and regular water.

Type of coating	Absorption rate (g/m^2^ day)	
	In saltwater	In regular water
MI	0.63 ± 0.04	0.70 ± 0.06
EP	0.02 ± 0.002	0.03 ± 0.002
BW	0.14 ± 0.05	0.25 ± 0.09
BR	0.07 ± 0.03	0.04 ± 0.02

MI coating samples obtained the highest absorption rate, which was between (0.54–0.76 g/m^2^ day) and 0.60–0.90 g/m^2^ day, followed by the BW coat with a range of (0.1–0.28 g/m^2^ day) and (0.15–0.67 g/m^2^ day) in regular water and in saltwater, respectively. The lowest absorption results were obtained in the case of EP and BR. EP coating presented values between (0.02–0.03 g/m^2^ day) in salty and regular water. BR coat showed values of (0–0.18 g/m^2^ day) and (0–0.11 g/m^2^ day) in salty and regular water. As expected, the materials that presented the best performance were the epoxy and the solvent-based coatings because of their low permeability properties, which minimize water penetration. On the other hand, mineral coating samples presented the highest absorption values caused by the presence of clinker Portland cement, which is characterized by increased concrete water absorption^30^ due to different salt combinations of sodium, potassium, and calcium^31^.

### Tensile test of the mineral coating

**Figure 14:**
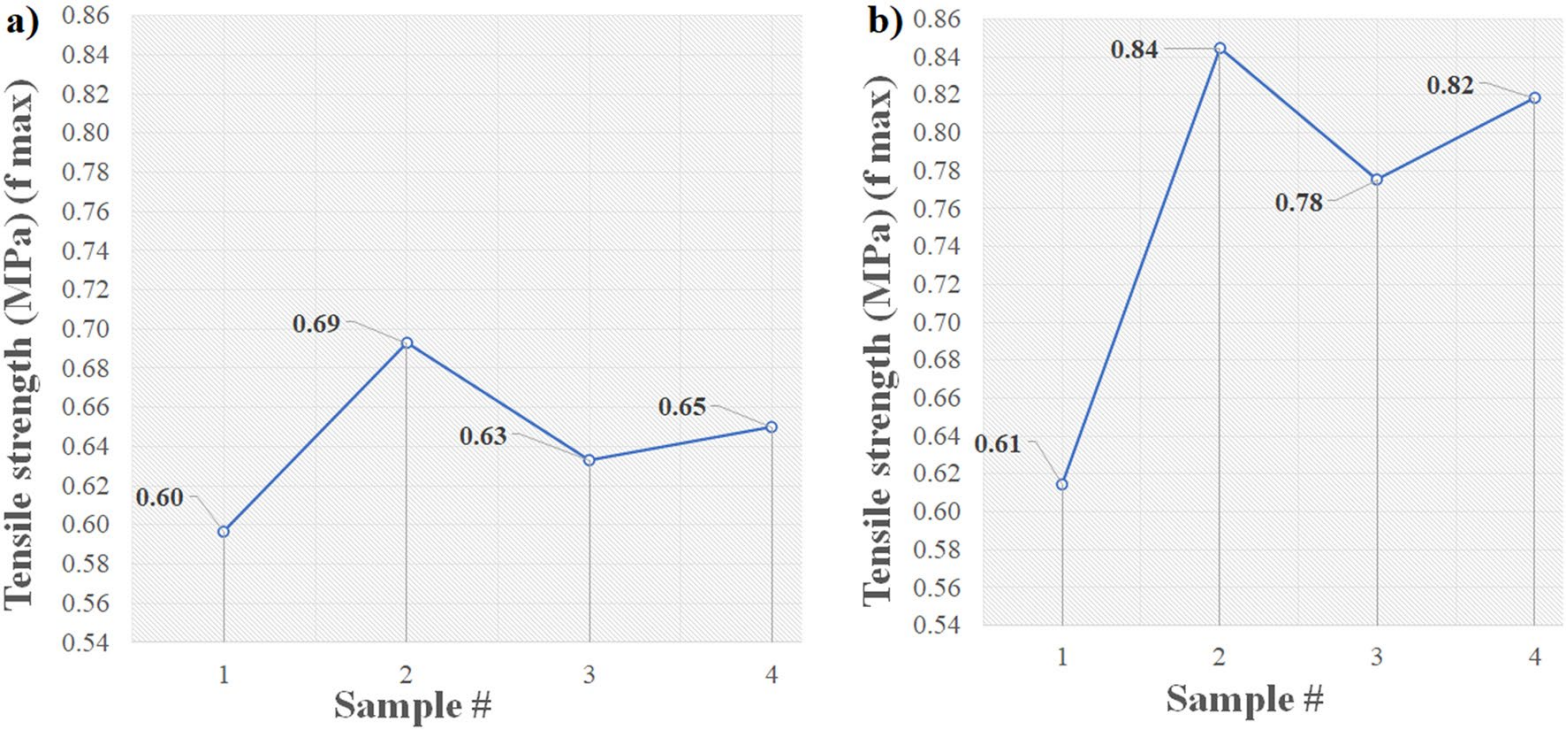
Tensile strength (**a**) after 14 days (**b**) after 28 days.

Figure 14 shows the tensile strength results at 14 and 28 days of mineral coating. It shows an increment of around 25% in their strength value over time.

**Figure 15:**
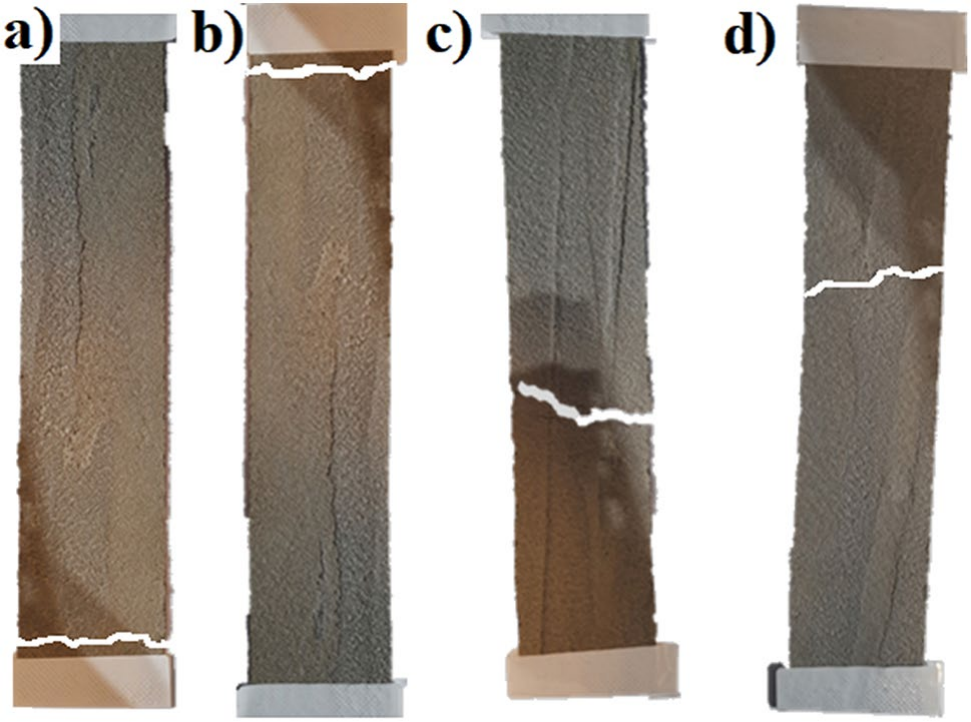
Detail failure mode and type: (**a**) Lateral at grip/tab Bottom (**b**) Lateral at grip/tab Top (**c**) Angled Gage Middle (**d**) Lateral Gage Middle.

After the test, samples were carefully photographed, and the failure type was determined according to the ASTM D 3039 standard. Figure 15 shows some examples of the typical failure modes obtained in this research.

Table 9 summarizes the failure modes, location, young modulus values, and the elongation of each sample.

**Table 9:** Summary of failure modes, young’s modulus, and elongation.

Sample #	Failure mode and location	Young’s modulus (Pa)	Elongation (%)
**After 14 days**			
1	Lateral at grip/tab top	3.09 E+0.7	1.93
2	Lateral at grip/tab top	3.58 E+0.7	1.93
3	Lateral at grip/tab top	3.29 E+0.7	1.92
4	Lateral at grip/tab bottom	3.36 E+0.7	1.93
**After 28 days**			
1	Lateral at grip/tab top	9.71 E+0.7	1.44
2	Lateral at grip/tab bottom	8.97 E+0.7	1.45
3	Angled gage middle	9.01 E+0.7	1.44
4	Lateral gage middle	8.97 E+0.7	1.45

The elastic modulus values of the MI coatings were evaluated after 14 and 28 days of air drying. The results for the samples dried during 14 days ranged from 30 to 35 MPa at room temperature and tripled their values after 28 days of drying, with a range of 90–97 MPa. These results demonstrate that the material increases its stiffness over time. In addition, the results were aligned with the manufacturer’s values. In the case of strain at breaking, the elongation results remained steady, showing a minimum variation among the results of 1% at 14 and 28 days at room temperature.

## Conclusions

The influence of salt and regular water and different types of drying methods has been studied and analyzed using four types of coating materials; mineral coating, epoxy coating, solvent-based coating, and water-soluble coating.

The study results have shown that the pull-off adhesion strength when the samples are air-dried for 28 days is reduced when the coating material is applied on an oily saturated surface. Mineral coating presented a dramatic fall of about 85% in its adhesion strength compared to 63% for water-soluble coatings and 35% for solvent-based coating. The presence of oil also affected the mineral coating, reducing about 83% of its strength when air or oven-dried. On the other hand, the pull-off adhesion strength increased by 20% for water-soluble coatings and 74% for solvent-based coatings when dried in the oven compared to the air-dried process. Based on this, it is possible to conclude that incorporating an oven-drying process increases the adhesion of the coating material to concrete by evaporating water and solvent residues.

During the exposition to salty and regular water, all samples showed changes in their physical appearance, presenting differences in color, manageability, and physical form. The presence of salts reduced water absorption mainly in the water-soluble specimens due to the non-hydrocarbon constituents presented in the material.

The epoxy coating was the only material not affected either by the drying method or by the presence of salts. EP showed the highest pull-off adhesion strength in both types of the drying processes, where the presence of oil or water did not impact the eventual results. In addition, this coating material presented the lowest absorption values among all samples, increasing its weight only by around 1% after 28 days of exposition.

It was evidenced that all coating materials improved the concrete resistance against carbonation by reducing carbonation depth at 90 days of standard curing. Based on the carbonation suppression ratio, the ranking order of the four types of coating material is EP > BW > BR > MI.

Finally, the tensile strength of MI coating demonstrates that the material’s stiffness increases over time; this is mainly because its the low water/cement ratio [W/C 0.20].

## Data Availability

All data generated or analyzed during this study that support the findings are available from the corresponding author upon reasonable request and with the permission of all authors.
